# *Helicobacter pylori* and colorectal cancer—A bacterium going abroad?

**DOI:** 10.1371/journal.ppat.1007861

**Published:** 2019-08-08

**Authors:** Julia Butt, Meira Epplein

**Affiliations:** Department of Population Health Sciences, Duke University and Cancer Control and Population Sciences Program, Duke Cancer Institute, Durham, North Carolina, United States of America; Tufts Univ School of Medicine, UNITED STATES

## Introduction

*Helicobacter pylori* is a bacterium that infects the mucus gel layer above the gastric epithelium in approximately half of the world’s population [1]. Infection with *H*. *pylori* causes gastritis, which may become chronic. Chronic inflammation of the stomach mucosa leads to morphological changes in the gastric epithelium transitioning from chronic atrophic gastritis to intestinal metaplasia, dysplasia, and, in 1%–3% of *H*. *pylori*–infected individuals, to gastric cancer [2]. This progression from normal to cancerous tissue takes decades and can be inhibited by eradication of the bacterium through antibiotic treatment [2]. Besides causing gastric cancer, which was officially acknowledged by the World Health Organization in 1994 [3], the presence of *H*. *pylori* has been associated with other diseases as well. Within the stomach, *H*. *pylori* increases risk of peptic ulcer and lymphoma of mucosa-associated lymphatic tissues; outside of the stomach, *H*. *pylori* has been found to be associated with not only a range of noncancerous diseases, including asthma, Parkinson, and diabetes, but also other cancerous outcomes in the gastrointestinal tract, particularly the colorectum [4, 5].

## Epidemiological findings

**Fig 1:**
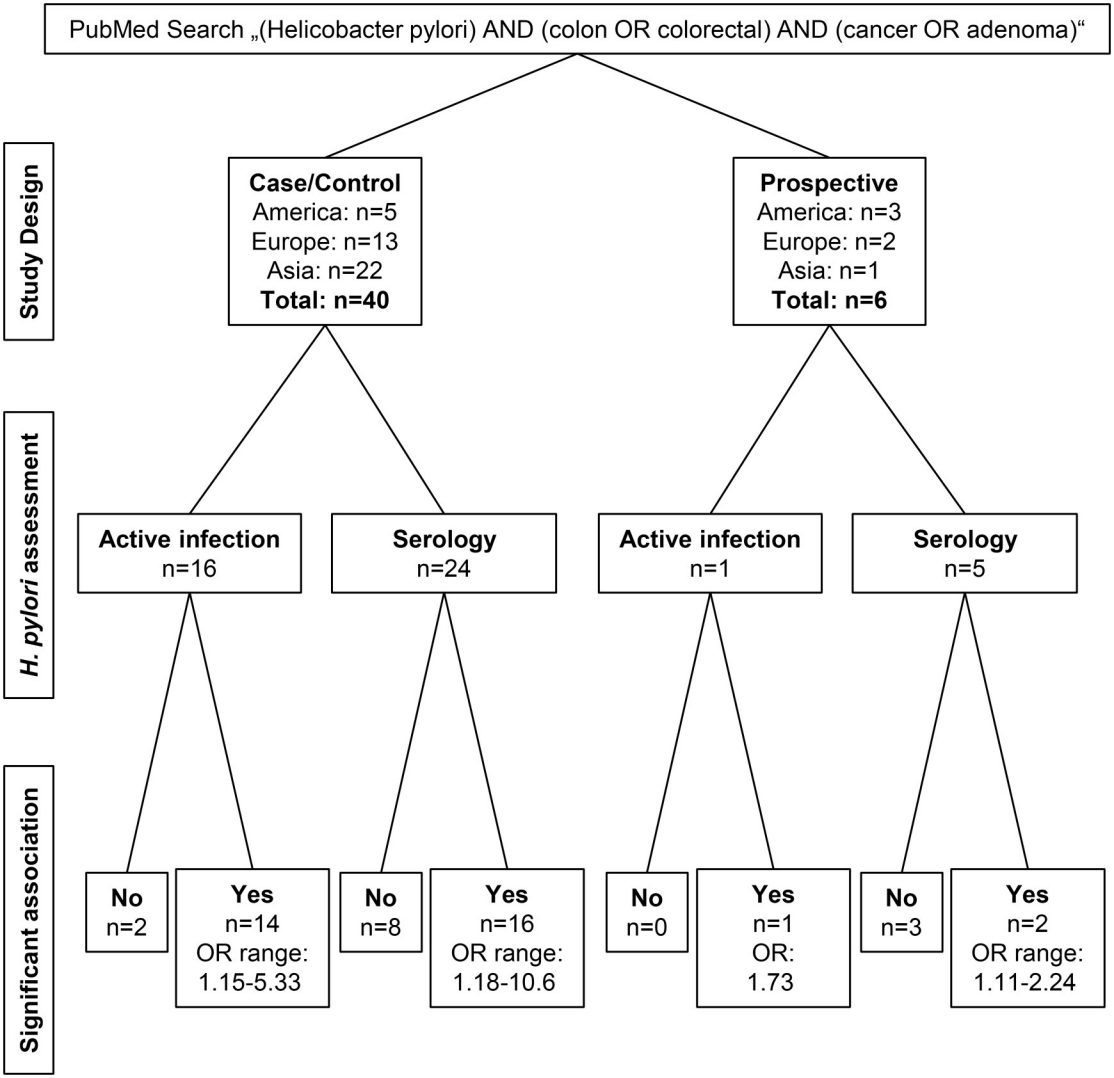
Original articles on *H*. *pylori* and colorectal adenoma and cancer published through March 2019 in PubMed. A PubMed literature search was performed to identify case-control and prospective studies assessing the association of *H*. *pylori* infection with colorectal adenoma and/or cancer. Identified studies varied by geographical region (America, Europe, Asia) and method to diagnose *H*. *pylori* infection (direct detection of active infection by urea breath test, gastric biopsy, and/or *H*. *pylori*–related gastric disease versus serology as an indirect measure of past and current infection).

Numerous studies have addressed the potential association of *H*. *pylori* infection with colorectal cancer (CRC) and precancerous lesions. In a PubMed search on the terms “(Helicobacter pylori) AND (colon OR colorectal) AND (cancer OR adenoma)”we found 40 case-control and six prospective studies (Fig 1) [6–51]. The majority of case-control studies (*n* = 22) were conducted in Asia, an area with a high burden of *H*. *pylori* infection [6, 7, 11, 18, 24–26, 28–33, 35, 39–42, 44, 46, 50, 51]. Screening programs for gastric cancer in certain Asian countries include esophagogastroduodenoscopy on a regular basis, which allows assessing *H*. *pylori* infection and severity of tissue damage directly from stomach biopsies to compare with the outcome of concomitant colonoscopies. This direct approach assesses active *H*. *pylori* infection, an advantage to serology. Independent of the method applied, however, the majority of case-control studies (*n* = 30) reported a positive association of *H*. *pylori* with colorectal adenoma and/or cancer prevalence, with odds ratios varying between 1.15 and 10.6 [6, 7, 9, 11, 12, 16–18, 21–35, 37–40, 42, 46, 51]. A meta-analysis by Wu and colleagues (2013) reported a summary estimate of 1.66 (95% confidence interval: 1.30–1.97) for colorectal adenoma and 1.39 (95% confidence interval: 1.18–1.64) for CRC [52], which reflects reported results from the majority of case-control studies cited in this review.

In the six prospective studies identified [10, 13, 14, 19, 20, 48], three were performed in the United States and analyzed samples from different races/ethnicities. Interestingly, among white populations, there was no increased risk of CRC with seropositivity to *H*. *pylori*, whereas significant increases in risk were reported among African Americans [10, 14, 20]. The serological method applied in these three studies was multiplex serology, an assay that allows the analysis of antibody responses to specific *H*. *pylori* proteins as opposed to general *H*. *pylori* seropositivity [53]. African Americans were reported to be at an approximately 2-fold increased risk of developing CRC with antibody responses to an *H*. *pylori* toxin, Vacuolating cytotoxin A (VacA) [10, 20]. This association was even reported with a dose–response relationship in terms of higher level of antibody response, which may signal a more severe infection with *H*. *pylori* in the stomach, being more strongly associated with CRC risk.

In summary, epidemiological findings in the literature hint toward a relation of *H*. *pylori* infection with CRC risk. However, whether this is a causal relationship and what the potential mechanisms are, as explained below, still need to be identified.

## Causality?—A question yet to be solved

As laid out above, the natural habitat of *H*. *pylori* infection is the stomach. Thus, the question that arises is whether the observed association of *H*. *pylori* with cancer in the colon could be due to a causal relationship. The positive associations in prospective studies suggest that reverse causality is not responsible for this association; however, it cannot be ruled out that other factors are the underlying reason for the reported observations. For example, *H*. *pylori* prevalence is generally associated with lower socioeconomic status worldwide [54, 55]. *H*. *pylori* infection is usually acquired in childhood, and living conditions during childhood, including household crowding, have been reported to be associated with *H*. *pylori* prevalence among adults [56]. The association observed between socioeconomic status and *H*. *pylori* in adults, however, could also relate to access to healthcare, a factor that also correlates with access to CRC screening. In our recently published study on *H*. *pylori* serology and CRC risk in diverse populations in the US, however, education, as a proxy for socioeconomic status, was not associated with risk of CRC and moreover did not confound the association of *H*. *pylori* with CRC, suggesting that low socioeconomic status is not a significant cause of the associations seen between *H*. *pylori* infection and increased CRC risk [10].

Intriguingly, CRC incidence and *H*. *pylori* prevalence coincide by population. From 2002 to 2012, an increase in CRC incidence was observed in China, Spain, and countries in Eastern Europe [57], countries that also report the highest *H*. *pylori* prevalence worldwide [1]. Furthermore, taking the US as an example, although the overall CRC incidence has been declining over the past decades, incidence rates are still 10%–20% higher in Alaskan Natives and African Americans as opposed to whites [58], and again, this coincides with a notable disparity in *H*. *pylori* prevalence [54]. As mentioned above, the observed associations were not confounded by educational level [10, 20]. Nevertheless, the association still may be affected by other cofactors including lifestyle, comorbidities, medications, and host response to the infecting bacterial strain [58, 59].

A recent study by Hu and colleagues (2018), conducted in Taiwan, further supports a causal relationship between persistent *H*. *pylori* infection and CRC development [60]. The authors follow up individuals with either no *H*. *pylori* infection, successful *H*. *pylori* eradication, or persistent *H*. *pylori* infection for the development of colorectal adenoma. The incidence rates of adenoma in the noninfected and eradicated group were comparable in the follow-up time period of 9 years, whereas the incidence rate in the group with persistent infection was 3-fold higher. Information on whether persistent infection was a consequence of antibiotic resistance, noncompliance to the applied antibiotic treatment, or reinfection during the study course was not further specified in the publication. Moreover, it would have been interesting to further define whether persistent *H*. *pylori* infection alone or together with associated epithelial damage in the stomach—such as atrophic gastritis, intestinal metaplasia, dysplasia, or even gastric cancer—was associated with colorectal adenoma incidence. And, as the authors conclude, the mechanisms of this potential causal relation still need to be elucidated.

## Potential mechanisms for a causal relationship

**Fig 2:**
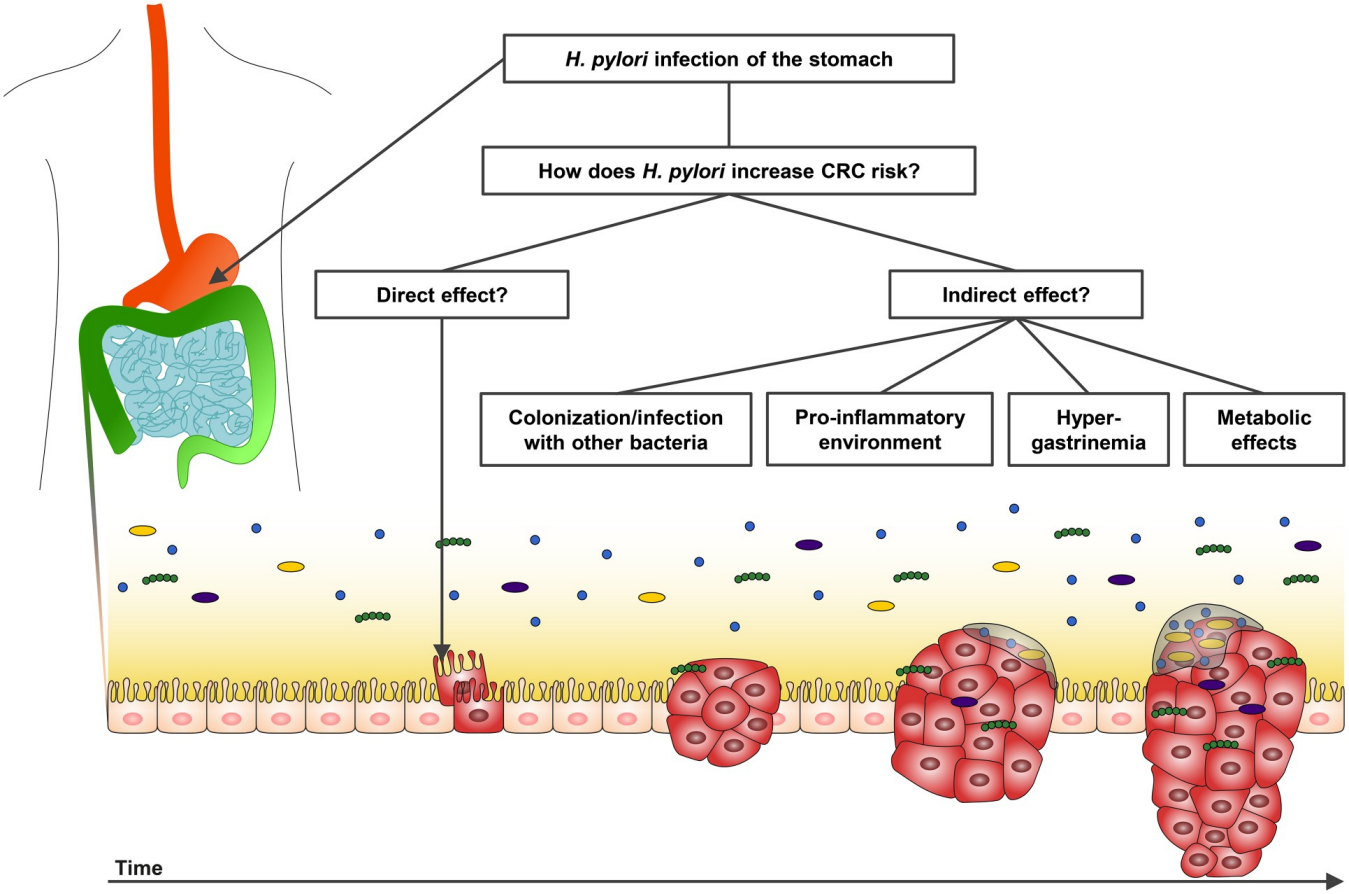
Potential mechanisms for causal effect(s) of *H*. *pylori* on colorectal carcinogenesis. The natural site of *H*. *pylori* infection is the stomach. Thus, the question arises how and when in the process from a healthy gut epithelium to CRC *H*. *pylori* might contribute to carcinogenesis. Besides a potential direct effect, *H*. *pylori* might exert indirect effects through enabling other bacteria to colonize/infect the colorectal epithelium and/or by causing systemic pro-inflammatory, hormonal, or metabolic changes. CRC, colorectal cancer.

If causal, *H*. *pylori* could have direct and/or indirect effects on colorectal carcinogenesis (Fig 2). A direct effect would involve the bacterium or molecular effectors, like secreted toxins, to be present in the respective colorectal tissue. Very few studies have addressed the presence of *H*. *pylori* in colorectal tumor tissue by PCR or histology [61–63]. Two case series found *H*. *pylori* in 22%–27% of analyzed colorectal polyps or cancers [61, 63]. A case-control study found positive *H*. *pylori* histology in tumor tissue of 19 out of 118 cases, whereas only one out of 58 controls were *H*. *pylori* tissue positive in the colon [62]. These findings plus the fact that a diagnostic test uses the presence of *H*. *pylori* antigen in stool lead to the assumption that *H*. *pylori* or constituents of it at least traverse the colon. Larger studies with normal tissue and tissue from all stages along the carcinogenic process in the colorectum are needed to confirm this hypothesis. It furthermore needs to be elucidated if and how *H*. *pylori* might then be able to elicit carcinogenic effects in the colorectum.

Under the assumption that *H*. *pylori* might not be able to infect the colorectal epithelium, there are several hypotheses as to how *H*. *pylori* could indirectly contribute to colorectal carcinogenesis. First, *H*. *pylori* infection could lead to changes in the colonization of the gut with other bacteria that in turn could contribute to colorectal carcinogenesis. A study by Gao and colleagues (2018), found that the gut microbiome composition did not differ between *H*. *pylori*–infected and –noninfected individuals but did differ by *H*. *pylori*–related gastric lesion—i.e., between *H*. *pylori*–infected individuals with normal, gastritis, and metaplastic tissue [64]. As described above, several case-control studies found higher odds for colorectal adenoma and cancer in individuals with *H*. *pylori*–related gastric lesion compared with healthy controls [11, 35, 39, 42].

Second, *H*. *pylori* infection could be involved in colorectal carcinogenesis by increasing the release of gastrin that may act as a mitogen. It has been shown that *H*. *pylori* induces hypergastrinemia; however, studies on the interplay of *H*. *pylori* infection, gastrin level, and CRC report inconclusive results and therefore need further investigation [15, 17, 23, 43, 44, 47]. Similarly, *H*. *pylori* was found to be associated with metabolic diseases that in turn were also described to be associated with CRC risk. The analyses of a combined effect of these with *H*. *pylori* on colorectal carcinogenesis are, however, also inconclusive. We found one case-control study that reported significant independent associations of metabolic syndrome and *H*. *pylori* with colorectal adenoma prevalence [32], as opposed to another study that found a combined increased association of *H*. *pylori* and diabetes with colorectal adenoma prevalence [6].

Finally, *H*. *pylori* induces chronic inflammation in the stomach mucosa, thereby also elevating systemically inflammatory responses in the body [65, 66]. Inflammation is reported to be associated with an increased CRC risk, and similarly, long-term intake of aspirin as an anti-inflammatory drug was shown to protect from CRC development [67]. It is unknown, though, whether *H*. *pylori* might create a pro-inflammatory state in the gastrointestinal tract that may increase CRC risk.

Comprehensive longitudinal studies are needed to address the interaction and temporality of the abovementioned potential mediators with the association of *H*. *pylori* and CRC risk. Furthermore, all of the abovementioned possibilities do not have to be exclusive mechanisms but, rather, most likely interact.

## Conclusion

There is growing evidence for *H*. *pylori* infection increasing CRC risk. A causal relationship and the mechanism behind this bacterium potentially acting abroad from its natural habitat, the stomach, however, still need to be clarified. If proven to be causal, with a direct and/or indirect pathway, *H*. *pylori* eradication might be an effective strategy to help prevent CRC development in a subset of cases. Independent of a causal relationship, the knowledge of the association of *H*. *pylori* and CRC risk could help to define more rigid CRC screening programs for *H*. *pylori*–positive individuals.
